# Expression of costimulatory molecules in the bovine corpus luteum

**DOI:** 10.1186/1477-7827-5-5

**Published:** 2007-01-31

**Authors:** Matthew J Cannon, John S Davis, Joy L Pate

**Affiliations:** 1 Department of Animal Sciences, The Ohio State University/Ohio Agricultural Research and Development Center, Wooster, Ohio 44691, USA; 2 Olson Center for Women's Health, Department of Obstetrics and Gynecology, University of Nebraska Medical Center, Omaha, Nebraska 68198; OVAMC 983255 Nebraska Medical Center, Omaha, Nebraska 68198, USA

## Abstract

**Background:**

Bovine luteal parenchymal cells express class II major histocompatibility complex (MHC) molecules and stimulate class II MHC-dependent activation of T cells in vitro. The ability of a class II MHC-expressing cell type to elicit a response from T cells in vivo is also dependent on expression of costimulatory molecules by the antigen presenting cell and delivery of a costimulatory signal to the T cell. Whether bovine luteal parenchymal cells express costimulatory molecules and can deliver the costimulatory signal is currently unknown.

**Methods:**

Bovine luteal tissue was collected during the early (day 5; day of estrus = day 0), mid (day 11–12), or late (day 18) luteal phase of the estrous cycle, and at 0, 0.5, 1, 4, 12 or 24 hours following administration of PGF2alpha to cows on day 10 of the estrous cycle. Northern analysis was used to measure CD80 or CD86 mRNA concentrations in luteal tissue samples. Mixed luteal parenchymal cell cultures and purified luteal endothelial cell cultures were prepared, and real-time RT-PCR was used to examine the presence of CD80 and CD86 mRNA in each culture type. Monoclonal antibodies to CD80 and CD86 were added to a mixed luteal parenchymal cell-T cell co-culture in vitro T cell proliferation assay to assess the functional significance of costimulatory molecules on activation of T lymphocytes by luteal parenchymal cells.

**Results:**

Northern analysis revealed CD80 and CD86 mRNAs in luteal tissue, with greatest steady-state concentrations at midcycle. CD80 and CD86 mRNAs were detected in mixed luteal parenchymal cell cultures, but only slight amounts of CD80 (and not CD86) mRNA were detected in cultures of luteal endothelial cells. Luteinizing hormone, PGF2alpha and TNF-alpha were without effect on concentrations of CD80 or CD86 mRNA in mixed luteal parenchymal cells cultures. Anti-CD80 or anti-CD86 monoclonal antibodies inhibited T cell proliferation in the in vitro T cell proliferation assay.

**Conclusion:**

It can be concluded from this study that parenchymal cells within the bovine CL express functional costimulatory molecules that facilitate interactions between with T cells, and these components of the antigen presentation pathway are expressed maximally in the midcycle CL.

## Background

The body of evidence implicating immune cells as regulators of luteal function is expanding. Macrophages and T lymphocytes are found in the corpus luteum (CL) of a number of species [1-9], as is messenger RNA and protein of several T cell-derived cytokines [5-10]. T cell cytokines such as interleukin-1β (IL-1β), tumor necrosis factor-α (TNF-α) and interferon-γ (IFN-γ) inhibit LH-stimulated steroidogenesis and induce PGF_2α _production in cultures of mixed luteal parenchymal cells [11-18]. Bovine luteal parenchymal cells express both class I and II major histocompatibility complex (MHC) molecules [19,20], which allow the cells to interact with CD8+ and CD4+ T lymphocytes, respectively. Expression of class II MHC *in vivo *increases near the time of luteal regression and in response to administration of a luteolytic dose of PGF_2α _[20]. Bovine luteal parenchymal cells also stimulate class II MHC-dependent proliferation of T lymphocytes *in vitro *[21,22], indicating that the class II MHC molecules expressed by luteal parenchymal cells are functional and that these cells can act as antigen presenting cells.

Class II-dependent presentation of antigen to T cells occurs via interaction of class II MHC molecules on the antigen presenting cell surface with the T cell receptor for antigen (TCR) on the T lymphocyte surface. With regard to T cells, there are two possible outcomes of MHC-mediated cellular interactions. In one instance, binding of MHC molecules to the TCR can occur in the absence of accompanying interactions between additional cell surface molecules. In this case, an inactive state known as anergy will be induced in the T cells [23-25]. Induction of anergy is one means by which tolerance to antigens in peripheral tissues is induced, thus avoiding an autoimmune response [26].

Alternatively, MHC-TCR ligation can occur in conjunction with costimulation. Costimulation is dependent on binding of costimulatory molecules present on the antigen-presenting cell to the lymphocyte receptor CD28. The two primary costimulatory molecules are CD80 and CD86, also referred to as B7-1 and B7-2 [27,28]. Binding of either costimulatory molecule to CD28 promotes T cell survival [29] and induces T cell activation and clonal expansion [30-32]. Therefore, depending on the presence or absence of costimulatory molecules on the antigen-presenting cell, MHC-mediated interactions have distinct and vastly different consequences. The objective of these studies was to determine whether luteal parenchymal cells express functional costimulatory molecules in order to understand whether the class II MHC-dependent interaction of luteal parenchymal cells with T lymphocytes induces anergy or activation of T cells.

## Methods

### Reagents

Powdered Ham's F-12 culture medium, gentamicin, fetal bovine serum, E. coli DH5α chemically competent cells, restriction enzymes and TRIzol Reagent were all purchased from Gibco/Life Technologies (Grand Island, NY). Recombinant murine TNF-α was purchased from Gibco/Life Technologies and R & D Systems. Bovine luteinizing hormone (LH; NIAMMD-bLH-4) was provided by the National Hormone and Pituitary Program (Baltimore, MD). Insulin-transferrin-selenium (ITS) premix was obtained from Collaborative Research Products. Bovine serum albumin (fraction V), prostaglandin (PG)F_2α_, and N-(2-hydroxyethyl) piperazine-N'-2-ethanesulfonic acid (HEPES) buffer were purchased from Sigma Chemical Co. (St. Louis, MO). Type I collagenase was acquired from Worthington Biochemical Corp. (Freehold, NJ). Sodium dodecyl sulfate (SDS) and 3-(N-morpholino) propanesulfonic acid (MOPS) buffer were acquired from Amresco (Solon, OH). Digoxigenin-labeled rNTP mix (10×), alkaline-phosphatase-conjugated anti-digoxigenin Fab fragments, CDP-Star chemiluminescent substrate, blocking reagent, and ampicillin were purchased from Roche Molecular Biochemicals (Mannheim, Germany). Recombinant RNasin, Moloney murine leukemia virus reverse transcriptase, *Taq*Bead FastStart polymerase, and T7 and T3 RNA polymerases were purchased from Promega (Madison, WI). Hybond N+ membranes were obtained from Amersham Pharmacia Biotech (Piscataway, NJ). Tissue culture flasks were from Corning (Corning, NY). All other chemicals were purchased from Sigma Chemical Co. or Fisher Scientific (Fair Lawn, NJ).

### Animals and tissue collection

Corpora lutea collected from normally cycling, multiparous, lactating dairy cows between three and six years of age were used in the present study. Corpora lutea to be used for RNA extraction for Northern analysis were cut into four equal pieces, snap frozen in liquid nitrogen, transported to the lab in liquid nitrogen, and stored at -80°C until RNA extraction was performed. Corpora lutea were collected at early (day 5, n = 4), mid (days 10–11; n = 4) or late (day 18; n = 4) stages of the estrous cycle, or at 0.5 (n = 4), 1 (n = 4), 4 (n = 4), 12 (n = 4) or 24 (n = 4) hours following i.m. administration of 25 mg of PGF_2α _(Lutalyse; Pfizer, New York, NY) on day 10 of the estrous cycle to induce luteal regression. In addition, lymph node tissue (used as a positive control) and skeletal muscle tissue (used as a negative control) were collected from cows at slaughter, snap frozen in liquid nitrogen, and stored at -80°C until RNA extraction.

Corpora lutea to be used for cell culture were removed on day 10 of the estrous cycle, immediately placed in ice-cold Ham's F-12 culture medium and transported to the laboratory for dissociation. Handling of animals and surgical procedures were carried out according to protocols approved by the Institutional Laboratory Animal Care and Use Committee of The Ohio State University.

### Dissociation of corpora lutea and culture of mixed luteal parenchymal cells

Dissociation of corpora lutea was carried out according to procedures described previously [33]. Cells derived from simple dissociation and culture of corpora lutea are referred to throughout as "mixed luteal parenchymal cells" to indicate the likely presence of steroidogenic luteal cells as well as some luteal endothelial cells in these cultures. Cell culture was performed in a humidified atmosphere of 5% CO_2 _in air at 37°C. Dispersed cells (4 × 10^6 ^cells/flask) were cultured in serum-coated 25 cm^2 ^flasks in a total of 4 ml of Ham's F-12 containing insulin (5 μg/ml), transferrin (5 μg/ml), selenium (5 ng/ml), gentamicin (20 μg/ml), and LH (1 ng/ml). Cells were allowed to adhere overnight, medium was replaced, and cultures were treated with LH (10 ng/ml), TNF-α (50 ng/ml), or PGF_2α _(10 ng/ml), in a 3 × 3 factorial arrangement, with all treatments performed in duplicate. The experiment was replicated a total of four times using CL from different animals. Treatment concentrations used in this study have been shown previously to affect function, viability, and gene expression in cultures of bovine mixed luteal parenchymal cells [15,34-36]. Medium and treatments were replaced after 24 hours. Total RNA was extracted from cultured cells after 48 hours of culture.

### Isolation and culture of purified luteal endothelial cells

Endothelial cells isolated from bovine CL collected during early pregnancy were purchased from Cambrex BioScience (BioWittaker, Walkersville, MD) as described previously [37-39]. These cells are referred to as "luteal endothelial cells" throughout, to distinguish them from cultures of mixed luteal parenchymal cells (see previous section). In the present study, luteal endothelial cells from frozen aliquots (passages 3–5) (5,000 cells/cm^2^) were cultured in EGM-2MV media, as recommended by the supplier with 3% fetal bovine serum (Cambrex Biosciences, BioWhittaker, Inc., Walkersville, MD) in 60 mm dishes. Cultures were maintained at 37°C in a humidified atmosphere of 5% CO_2 _and 95% air. Culture medium was replaced every 48 hours until 80–90% confluent. On the day of harvest, cells were equilibrated for 2–3 hr in serum-free EBM-2 medium. Luteal endothelial cells were collected in RLT lysis buffer (guanidine isothiocyante buffer) from the Qiagen RNeasy kit, and RNA was isolated using the RNeasy kit (Qiagen, Valencia, CA) according to the manufacturer's specifications.

### RNA isolation and northern analysis

Frozen luteal tissue was homogenized in TRIzol reagent using a Polytron tissue homogenizer (Brinkman Instruments, Westbury, NY). For extraction of total RNA from cultured cells, TRIzol reagent was added directly to individual culture flasks, and samples from each culture flask were isolated separately (samples were not pooled). Following tissue homogenization or cell lysis, total cellular RNA was isolated according to the TRIzol specifications sheet. The final RNA precipitate was resuspended in DEPC-treated double-distilled water, and RNA concentration was determined spectrophotometrically.

Bovine CD80 and CD86 have cDNA sequences have been described (GenBank accession numbers Y09950 and AJ291475[40]). Plasmids containing these sequences were kindly provided by Dr. Keith Parsons (Institute for Animal Health, Berkshire, UK), and were used to synthesize riboprobes. Glyceraldehyde-3-phosphate was used to normalize signals from CD80 and CD86 in Northern analysis, and a partial cDNA fragment of bovine dehydrogenase (GAPDH) was generated by RT-PCR using bovine-specific primers described previously [41]. This fragment was inserted into the pGEM-T Easy Vector (Promega), and the identity confirmed by sequencing. Anti-sense digoxigenin-labeled riboprobes were generated using linearized plasmids, according to the instructions in Roche Molecular Biochemicals' DIG Nonradioactive Nucleic Acid Labeling and Detection System manual. Corresponding sense riboprobes were also synthesized and were used as controls to confirm riboprobe specificity.

For Northern analysis, PolyA^+ ^RNA was isolated from total cellular RNA using the PolyATract mRNA Isolation System (Promega) according to the manufacturer's specifications. One microgram of polyA^+ ^RNA was electrophoretically separated on 1.5% agarose denaturing gels containing 20 mM MOPS, 5 mM sodium acetate, 1 mM EDTA and 0.66 M formaldehyde. Transfer of RNA to Hybond-N+ membranes was carried out using 10 × SSC (1.5 M NaCl, 0.15 M Na_3_C_6_H_5_O_7_·2H_2_O). Transfers were allowed to proceed for approximately 20 hours. Following transfer, membranes were baked at 80°C for 2 hours to crosslink RNA to the membranes.

Prehybridization and hybridization were performed in a Hybaid Micro-4 hybridization oven (ThermoHybaid, Franklin, MA). Membranes were prehybridized at 68°C for one hour with hybridization core buffer [250 mM Na_2_HPO_4_, 1 mM EDTA, 5% (w/v) SDS, 0.5% (w/v) blocking buffer]. Simultaneous hybridization was performed using either CD80 and G3PDH riboprobes or CD86 and G3PDH riboprobes. Riboprobes were denatured for 15 minutes at 68°C in 200 μl of hybridization buffer prior to use in hybridizations. After 1 hour of prehybridization, buffer was replaced, denatured riboprobes were added, and hybridization was carried out for 16 hours at 68°C. Following hybridization, membranes were washed and bound DIG-labeled riboprobes were detected using alkaline phosphatase-conjugated anti-DIG Fab fragments (1:20,000) in concert with chemiluminescent CDP-Star substrate. Membranes were exposed to Biomax ML film (Eastman Kodak Co. Rochester, NY) to detect chemiluminescence.

To quantify steady state concentrations of RNA for each message, densitometry was performed using a PDI 420oe Scanning Densitometer. The densitometric values (in arbitrary units) of each band were normalized to the densitometric values of the corresponding G3PDH band, to correct for loading inconsistencies.

### Reverse-transcription polymerase chain reaction

Sequences, annealing conditions, GenBank accession numbers for corresponding targets, amplicon sizes, and references [40-43] for primer sequences used in all RT-PCR are listed in Table 1. Primer sets were tested in luteal tissue samples to confirm amplification of single bands, amplified products were cloned and sequenced to confirm their identity, prior to use of primers in analysis of samples.

**Table 1:** 

Target	Primer Sequence	Amplicon Size	Annealing Temp (°C)	GenBank Accession no.	Reference
CD80	Forward: 5'-GAACCGCACCATCACTGACA-3' Reverse: 5'-TAATGGTCCAGGTCAGGTGC-3'	485 bp	56°C	Y09950	[40]
CD86	Forward: 5'-GACCTTGAGACTCCACAACG-3' Reverse: 5'-GTAGAGCTGCAATCCAGAGG-3'	490 bp	58°C	AJ291475	-
CD31	Forward: 5'-GTTCAGCGAAGTTCTGCGAG-3' Reverse: 5'-CTTGCTGGCTGTGGTCTTGT-3'	229 bp	58°C	U35433	[43]
GAPDH	Forward: 5'-AAGATTGTCAGCAATGCC-3' Reverse: 5'-ACAGACACGTTGGGAG-3'	293 bp	56°C	BC102589	-
GAPDH	Forward: 5'-TGTTCCAGTATGATTCCACCC-3' Reverse: 5'-TCCACCACCCTGTTGCTGTA-3'	854 bp	58°C	NM_001034034	[41]
StAR	Forward: 5'-CCTCTCTACAGCGACCAA-3' Reverse: 5'-TCGTGAGTGATGACCGTG-3'	311 bp	58°C	Y17259	[42]

A semi-quantitative RT-PCR assay was used to determine steady-state concentrations of CD80 mRNA in cultures of bovine mixed luteal parenchymal cells treated with LH, PGF_2α _and/or TNF-α. Amplification of CD80 and GAPDH was performed in duplicate parallel reactions. In preliminary experiments, the number of cycles in which the reactions entered the logarithmic phase of amplification was determined individually for each primer set by performing serial reactions with a single sample, incrementally increasing the number of cycles, and determining empirically the cycle number at which amplification is in the logarithmic phase. In these experiments, G3PDH amplification was in the logarithmic phase at 25 cycles, whereas CD80 was in logarithmic phase at 32 cycles. Reverse transcription was performed on 200 ng of total RNA for 15 minutes at 42°C using M-MLV reverse transcriptase. Following reverse transcription, thermal cycling was performed under the following conditions: denaturing, 94°C for 30 seconds; annealing (temperatures listed in Table 1) for 30 seconds; extension, 72°C for 60 seconds. Following amplification, PCR products were separated on 1.5% agarose gels and visualized with ethidium bromide. Densitometric values (in arbitrary densitometric units) of CD80 bands were normalized to values of corresponding G3PDH bands prior to statistical analysis.

Steady-state concentrations of CD86 mRNA in cultures of mixed luteal parenchymal cells treated with LH, PGF_2α _and/or TNF-α were determined by quantitative PCR analysis using a Roche LightCycler real-time PCR thermal cycler. Reverse transcription on 200 ng of total cellular RNA, as described above for semi-quantitative RT-PCR. Quantitative PCR was then performed using 40 ng of reverse transcribed total RNA, with the Roche FastStart DNA Master SYBR Green I kit according to the manufacturer's instructions. Ramping speed for transition from denaturing to annealing steps and from annealing to extension step was slowed to 1°/sec to mimic conditions in a block-type thermal cycler, while ramping speed from extension to denaturing steps was set at 20°/sec to minimize total running time. Thermal cycling was carried out using the following conditions: denaturing, 94°C for 30 seconds; annealing (temperatures listed in Table 1) for 30 seconds; extension, 72°C for 60 seconds, 40 cycles total. As a standard curve, the bovine CD86 insert provided by Dr. Keith Parsons was removed from the pCDNA3 vector by digestion with BamHI and EcoRI. The insert was electrophoretically separated, eluted from the gel, and the concentration of insert DNA was determined spectrophotometrically. The CD86 insert was then used to prepare serial dilutions to be used as an external standard curve in the quantitative PCR assays. Melting curve analysis performed during validation of the primers revealed the presence of a minor primer dimer product in the CD86 amplification reactions, with a melting temperature of approximately 75°C. Therefore, fluorescence was measured in both samples and standards at 77°C, to denature primer dimer products and eliminate any fluorescence due to binding of SYBR Green to these undesired products. Fluorescence values were measured in each reaction at the end of each cycle using the single acquisition mode. A melting curve analysis was performed after the end of the last cycle in order to verify the amplification of a single product in each sample. Samples were run in duplicate and fluorescence values for duplicate samples were averaged. The baseline fluorescence reading and noise band cut-off were set manually for samples and standards, in order to eliminate background fluorescence values from calculation of the slope and y-intercept of the standard curve. GAPDH was amplified in duplicate parallel reactions, and the average of the duplicate CD86 values from each sample was normalized to the corresponding average of the duplicate GAPDH values for each sample. A standard curve was generated using average fluorescence values of duplicate standards, and average fluorescence values of samples were then used to calculate the concentration of CD86 mRNA in each sample, using the Fit Points methods of the accompanying Light Cycler Data Analysis software.

Steady-state concentrations of mRNA encoding StAR, CD31, CD80, CD86 and GAPDH were determined in total RNA extracted from cultures of mixed luteal parenchymal cells and luteal endothelial cells, using an MJ Research Opticon 2 real-time PCR thermal cycler. StAR (a marker for steroidogenic cells) and CD31 (a marker for endothelial cells) mRNAs were amplified as a means to assess the composition of cultures of mixed luteal parenchymal cells and luteal endothelial cells. Prior to PCR, reverse transcription using random hexamer primers was performed on 2 μg of total RNA extracted from cultures. PCR was then performed, using 200 ng of reverse transcribed cDNA, using the DyNAmo™ HS SYBR Green qPCR kit according to the manufacturer's instructions. Thermal cycling was carried out using the following conditions: denaturation, 94°C for 30 seconds; annealing, (temperatures listed in Table 1) for 30 seconds; extension, 72°C for 60 seconds, for a total of 32 cycles. Melting curve analysis was performed after the end of the last cycle, and in conjunction with gel electrophoresis of amplified products, was used to verify the amplification of a single product in each sample. Fluorescence values in each tube were measured at the end of each cycle using single acquisition mode. Fluorescence values of the product of interest in each sample were standardized to the corresponding GAPDH fluorescence values, and these standardized values were then used to calculate the mean steady-state amounts of each message. Since no standard curve was run for the messages of interest, values were expressed in arbitrary units of fluorescence for purposes of analysis.

### Isolation of T lymphocytes

Isolation of T lymphocytes from whole blood was performed as described previously [22]. Briefly, blood was collected from cows via jugular venipuncture at the time of CL removal. Acid citrate dextrose (ACD) solution (41.6 mM citric acid, 74.8 mM sodium citrate, 136 mM dextrose) was used to prevent coagulation of whole blood (15 ml of ACD per 85 ml of whole blood). Anticoagulated whole blood was centrifuged, white blood cell layers were collected and centrifuged through Ficoll-Hypaque to isolate peripheral blood mononuclear cells (PBMC). T lymphocytes were separated from PBMC by depletion of class II MHC-positive cells using the mouse anti-bovine monoclonal antibodies TH14B, TH81A5, and H42A (VMRD, Pullman, WA) in conjunction with the MACS Cell Separation System (Miltenyi Biotec, Inc. Auburn, CA). This separation procedure yielded a population of cells that is approximately 96% positive for CD3 (as determined by indirect immunofluorescence using anti-CD3 antibody MM1A [VMRD]).

### Co-culture of luteal cells and T lymphocytes

Co-culture of mixed luteal parenchymal cells and T cells was performed as described previously [21,22]. Briefly, mixed luteal parenchymal cells were treated with 50 μg/ml mitomycin C to prevent proliferation of the cells in culture, and 3.2 × 10^4 ^mixed luteal parenchymal cells were placed in culture with 1.0 × 10^5 ^T lymphocytes in 96-well plates (Corning, Corning, NY). Cultures were performed in RPMI 1640 containing 10% heat-inactivated fetal calf serum, 25 mM HEPES, 2 mM L-glutamine, 100 IU penicillin and 100 μg/ml streptomycin in the presence of 1 μg/ml staphylococcal enterotoxin B (SEB). T lymphocytes were also cultured with mitomycin C-treated PBMCs, which contain antigen presenting cells such as monocytes and B lymphocytes. These cultures served as a positive control to determine the effects of antibody treatments.

To determine the importance of costimulation to the activation of T lymphocytes by bovine mixed luteal parenchymal cells, co-cultures were treated with monoclonal antibodies raised against bovine CD80 or CD86. Antibodies (murine ascites fluid diluted 1:10 with PBS containing 0.1% NaN_3_) were the generous gift of Dr. Keith Parsons, and were dialyzed exhaustively against PBS using Slide-A-Lyzer dialysis cassettes (Pierce, Rockford, IL). Dialyzed antibodies were added to co-cultures at a final treatment concentration equivalent to a 1:1000 dilution of ascites fluid.

All co-cultures were performed in a humidified atmosphere of 5% CO_2 _in air at 37°C for 72 hours. During the last 6 hours of culture, 0.5 μCi ^3^H-thymidine was added to each well to measure cellular proliferation. At the end of the 72 hour culture period, culture plates were frozen at -80°C. Cells were subsequently harvested using a semi-automatic cell harvester (Skatron Instruments, Sterling, VA) and incorporation of ^3^H-thymidine into the cellular DNA was determined by scintillation counting.

### Statistical analysis

For Northern analyses, differences in normalized densitometric values of CD80 and CD86 mRNA between stages of the estrous cycle and following administration of PGF_2α _were determined using one-way analysis of variance. Similarly, for semi-quantitative RT-PCR analysis of CD80 mRNA in cultured cells, normalized densitometric values of CD80 cDNA were subjected to one-way ANOVA to determine whether differences existed between culture treatment means. Relative differences in steady-state concentrations of CD31, StAR, CD80 and CD86 mRNA between cultures of mixed luteal parenchymal cells or luteal endothelial cells were determined using Student's T-test. Steady-state concentrations of CD86 mRNA in cultures of mixed luteal parenchymal cells treated with LH, PGF_2α _and/or TNF-α determined by quantitative RT-PCR analyses were subjected to one-way ANOVA to determine if differences existed between culture treatment means. In all ANOVA analyses, the Student-Newman-Keuls (SNK) test was used to determine differences between specific means. Differences in mean cell counts from co-culture experiments were also determined using one-way ANOVA, and the SNK test was used to determine differences between specific means. Means were considered significantly different at p < 0.05. Statistical analyses were performed using the SigmaStat statistical analysis software package (Jandel Corporation, San Rafael, CA).

## Results

**Figure 1:**
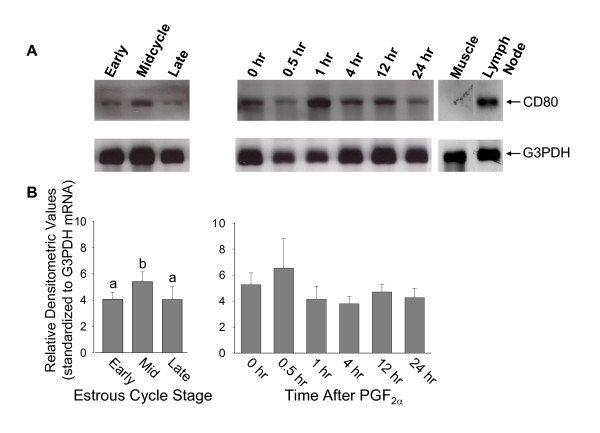
Northern analysis of CD80 and bovine luteal tissue. A) Representative Northern blot of CD80 mRNA and corresponding G3PDH mRNA in luteal tissue samples collected early, during midcycle, or late in the estrous cycle, and at 0, 0.5, 1, 4, 12 or 24 hours following administration of PGF_2α_. Muscle tissue RNA served as a negative control, and lymph node RNA served as a positive control. B) Steady-state concentrations of CD80 mRNA in luteal tissue. Bars represent densitometric values of CD80 mRNA normalized to G3PDH mRNA detected by Northern analysis (n = 4 CL at each time point). Values with different letters tended to be different (p = 0.074).

Northern analysis revealed the presence of CD80 and CD86 mRNA in luteal tissue throughout the estrous cycle and at all time points during PGF_2α_-induced luteal regression. In these studies, lymph node tissue, which contains antigen-presenting cells such as macrophages, dendritic cells and B lymphocytes, was used as a source of positive control RNA. Muscle tissue was used as a source of negative control RNA. Figure 1 displays the results of Northern analysis of CD80 mRNA in luteal tissue. A single band representing a transcript of approximately 3.0 Kb was present in positive control RNA, and a corresponding band was also present in all luteal tissue samples (Figure 1a). Muscle tissue RNA, which served as a negative control, was devoid of any transcript. Mean densitometric values representing steady-state concentrations of CD80 mRNA in the CL (n = 4 CL per time point) are displayed in Figure 1b. Steady-state concentrations of CD80 mRNA were not significantly different (p > 0.05), but tended to be greater (p = 0.74) in CL collected at midcycle compared with CL collected either early or late in the estrous cycle (Figure 1b). There were no changes in CD80 mRNA concentrations in response to administration of PGF_2α _to the cow (p > 0.10).

**Figure 2:**
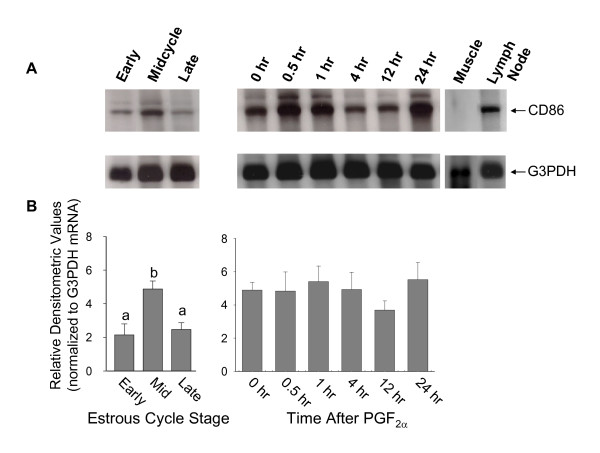
A) Representative Northern blot of CD86 mRNA and corresponding G3PDH mRNA in luteal tissue samples collected early, during midcycle, or late in the estrous cycle, and at 0, 0.5, 1, 4, 12 or 24 hours following administration of PGF_2α_. Muscle tissue RNA served as a negative control, and lymph node RNA served as a positive control. B) Steady-state concentrations of CD86 mRNA in luteal tissue. Bars represent densitometric values of CD86 mRNA normalized to G3PDH mRNA detected by Northern analysis (n = 4 CL at each time point). Different letters indicate significant differences (p < 0.05).

Results of Northern analysis of CD86 mRNA in luteal tissue are displayed in Figure 2. A single band representing a transcript of approximately 3.1 Kb was present in RNA from lymph node tissue. A corresponding band was also present in RNA from luteal tissue (Figure 2a). There was a second, less abundant band of greater size present in luteal tissue, to which there was no corresponding band in positive control RNA. Since there are no reports in the literature of a second larger transcript of CD86 and we are uncertain of the identity of this band, it was excluded from analyses. Muscle tissue RNA (negative control) was devoid of any transcript. Figure 2b depicts densitometric values representing the mean steady-state concentrations of CD86 mRNA in the CL (n = 4 CL per time point). In CL collected during the estrous cycle, highest concentrations of CD86 mRNA were present in midcycle CL (p < 0.05) as compared to early or late CL. Similar to CD80 mRNA, *in vivo *administration of PGF_2α _did not affect the concentrations of CD86 mRNA present in luteal tissue (p > 0.10).

**Figure 3:**
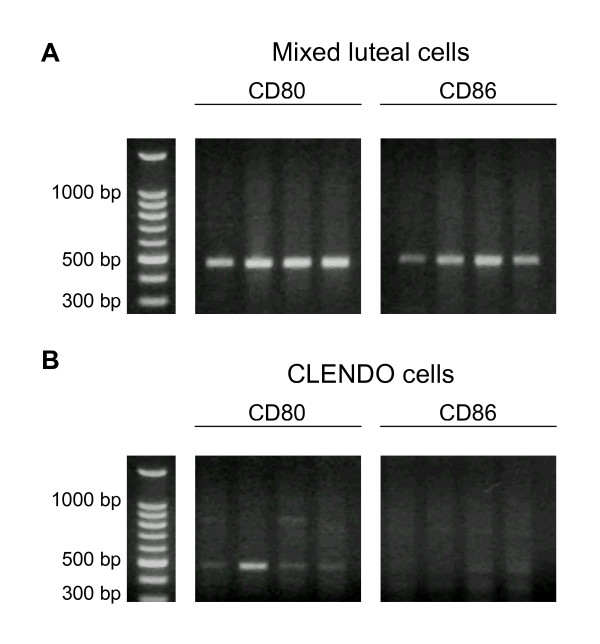
Agarose gel displaying PCR products from quantitative RT-PCR analysis of CD80 and CD86 mRNA in samples extracted from cultures of (A) mixed luteal cells or (B) luteal endothelial (CLENDO) cells (n = 4).

To address the possibility that endothelial cells of the CL express costimulatory molecules, the presence and steady-state concentrations of CD80 and CD86 mRNA were assessed in cultures of highly enriched luteal endothelial cells. To determine the composition and purity of the mixed luteal parenchymal cell and luteal endothelial cell cultures, quantitative RT-PCR to determine steady-state concentrations of CD31 (an endothelial cell adhesion molecule) and steroidogenic acute regulatory protein (StAR) mRNAs was performed. Concentrations of CD31 mRNA were approximately 10-fold greater (p > 0.05) in cultures of luteal endothelial cells compared with mixed luteal parenchymal cell cultures (data not shown). StAR mRNA was undetectable in some luteal endothelial cultures, and was present in concentrations near the detection limit of the PCR assay in others, but was present in high concentrations in cultures of mixed luteal parenchymal cells (data not shown). CD80 and CD86 mRNA was present and easily detectable by quantitative RT-PCR in mixed luteal parenchymal cell cultures (Figure 3a). In contrast, only CD80 mRNA was present in luteal endothelial cell cultures (Figure 3b), but steady-state concentrations of CD80 mRNA were much less in luteal endothelial cell cultures compared with mixed luteal parenchymal cell cultures (data not shown).

**Figure 4:**
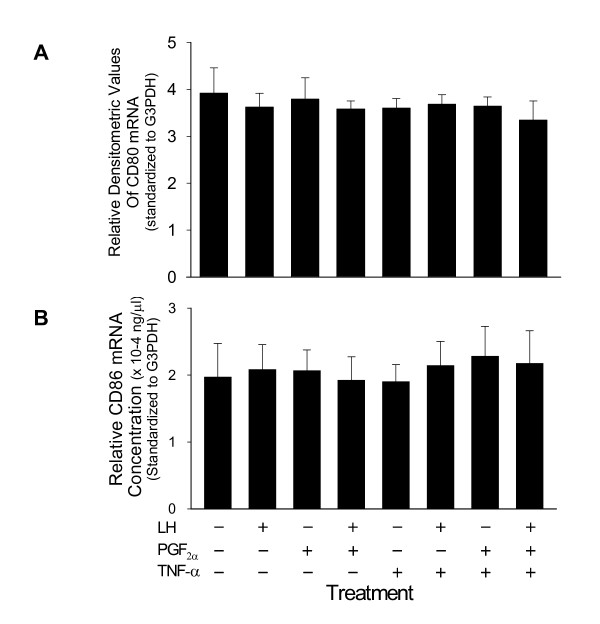
PCR analysis of regulation of CD80 and CD86 expression in cultured luteal cells. Bars represent steady-state concentrations of CD80 and CD86 mRNA in cultured bovine luteal cells treated with LH (10 ng/ml), PGF_2α _(10 ng/ml), and/or TNF-α (50 ng/ml). A) Bars represent densitometric values of amplified CD80 mRNA products, standardized to G3PDH, as detected by semi-quantitative RT-PCR analysis. There were no significant differences (p > 0.10; n = 4). B) Bars represent CD86 cDNA concentrations, as determined by RT-qPCR analysis. There were no significant differences (p > 0.10; n = 4).

To examine the regulation of costimulatory molecule expression, cultures of mixed luteal parenchymal cells were treated with 10 ng/ml LH, 10 ng/ml PGF_2α_, or 50 ng/ml TNF-α, and all possible combinations of each. Due to the limited amount of total RNA isolated from individual cultures, RT-PCR analysis was used to examine relative steady-state concentrations of CD80 and CD86 mRNA in cultures of mixed luteal parenchymal cells. We wished to use quantitative real-time RT-PCR (RT-qPCR) to measure both transcripts, however we were unable to develop a working primer set corresponding to CD80 for use in the Roche LightCycler. Therefore, semi-quantitative RT-PCR was used to determine steady-state concentrations of CD80 mRNA in mixed luteal parenchymal cell cultures, whereas RT-qPCR was used to determine concentrations of CD86 mRNA. Analysis of steady-state concentrations of CD80 mRNA revealed the presence of a 485 bp amplified product corresponding to CD80 mRNA in all mixed luteal parenchymal cell cultures (not shown). There were no changes in relative concentrations of CD80 mRNA associated with any treatments used in this study (Figure 4a). Similarly, RT-qPCR analysis showed no changes in steady-state concentrations of CD86 mRNA in cultures of mixed luteal parenchymal cells, regardless of treatment (Figure 4b).

**Figure 5:**
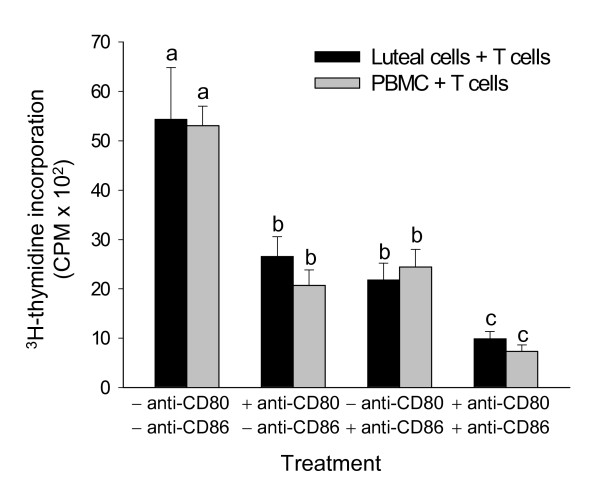
Effects of anti-CD80 and anti-CD86 antibodies on luteal cell-stimulated T lymphocyte proliferation. Bars represent T lymphocyte proliferation (as measured by H^3^-thymidine incorporation) in co-cultures of luteal cells and T lymphocytes (black bars) or PBMCs and T lymphocytes (gray bars), in the absence or presence of anti-CD80 or anti-CD86 mAbs. Difference superscripts represent significant differences (n = 4; p < 0.05).

T lymphocyte proliferation assays were performed to determine whether costimulatory molecules were functional participants in interactions between mixed luteal parenchymal cells and T cells *in vitro*. The results of this experiment are depicted in Figure 5. T cell proliferation (as measured by ^3^H-thymidine incorporation) in co-cultures of mixed luteal parenchymal cells and T lymphocytes was inhibited in the presence of anti-CD80 or anti-CD86 monoclonal antibody alone (p < 0.05), and T cell proliferation was further inhibited when both antibodies were used in combination (p < 0.05). Addition of anti-CD80 or anti-CD86 to cultures containing PBMCs (which contain antigen presenting cells such as monocytes/macrophages and B cells) and T lymphocytes had a similar effect.

## Discussion

To the best of our knowledge this study is the first in which expression of CD80 and CD86 in luteal tissue of any species has been reported. We have also demonstrated in this study that cells of the corpus luteum express functional costimulatory molecules that facilitate interactions between luteal parenchymal cells and T lymphocytes, enhancing the ability of mixed luteal parenchymal cells to stimulate T lymphocyte activation (as measured by proliferation) *in vitro*. Northern analyses revealed that the greatest steady-state concentrations of mRNA for both costimulatory molecules are present in luteal tissue at midcycle. Macrophages present in luteal tissue are likely to be one source of costimulatory molecule mRNA in the CL, since these cell types express costimulatory molecules [44,45]. In the cow, the number of macrophages present in the CL is higher during the mid (day 12 post-estrus) and late (day 18 post-estrus) luteal phase compared with the early luteal phase (day 6 post-estrus) [6]. Northern analysis in the present study revealed a similar trend with regard to concentrations of CD80 and CD86 mRNA in luteal tissue collected during the early and mid luteal phase.

In addition, CD80 mRNA, but not CD86 mRNA, was found in cultures of highly enriched luteal endothelial cells. With regard to the luteal endothelial cell cultures used in the present study, these cultures appear almost completely devoid of luteal cells expressing StAR (steroidogenic luteal cells). The concentration of mRNA encoding the endothelial cell adhesion molecule CD31 in luteal endothelial cell cultures was approximately 10-fold greater than in mixed luteal parenchymal cell cultures, whereas StAR mRNA was nearly undetectable. The mixed luteal parenchymal cell cultures used in the present study contain largely steroidogenic cells, but are likely to also contain small numbers of endothelial cells; StAR mRNA was abundant in the mixed luteal parenchymal cell cultures, but concentrations of CD31 mRNA were much less than those in luteal endothelial cell cultures. It is possible that significant numbers of macrophages are present in mixed luteal parenchymal cell cultures, and these cells could account for the greater concentration of CD80 mRNA present in mixed luteal parenchymal cell cultures compared with luteal endothelial cell cultures, as well as the presence of CD86 mRNA in mixed luteal parenchymal cell cultures but not in luteal endothelial cell cultures. It seems unlikely that macrophages are present in the luteal endothelial cell cultures, due to the purification procedures used to derive these cultures. However, the possibility that a small number of contaminating macrophages are the source of CD80 mRNA detected in these cultures by RT-PCR cannot be excluded. The absence of CD86 mRNA and very low amounts of CD80 mRNA were unexpected and surprising, since we have detected high concentrations of mRNA for class II MHC molecules in luteal endothelial cell cells (Cannon et al., unpublished). A pure population of steroidogenic luteal cells (ie cells free of macrophages and endothelial cells) could not be obtained, and therefore it is not possible to conclude from the present studies whether or not steroidogenic luteal cells express CD80 and CD86. However, it is evident from this portion of the study that endothelial cells of the corpus luteum do not express CD86. Luteal endothelial cells may express CD80, but the possibility that small numbers of contaminating macrophages are the source of CD80 mRNA in luteal endothelial cell cultures cannot be ruled out at this time.

The *in vitro *co-culture experiments performed in this study demonstrate that costimulatory molecules are expressed on cells of the bovine CL are functional, since antibodies against these cell surface proteins inhibit luteal parenchymal cell-stimulated T cell proliferation. Culture medium containing 10% fetal bovine serum is employed in these experiments in conjunction with mixed luteal parenchymal cells. Despite routine precautions taken during the dissociation procedure to minimize the presence of endothelial cells and macrophages, it is possible that, in addition to steroidogenic luteal cells, these cell types are also present in the co-cultures of mixed luteal parenchymal cells and T cells. We have demonstrated that CD80 mRNA is present in cultured luteal endothelial cells, but we have been unable to determine immunohistochemically the identity of the cells within the CL that express CD80 and CD86. The monoclonal anti-bovine CD80 and CD86 antibodies used in our co-culture experiments were not able to detect costimulatory molecules in control tissues (lymph node, spleen and liver tissue) or luteal tissue. Additionally, polyclonal anti-human anti-CD80 and anti-CD86 antisera available commercially from R&D systems were employed in further attempts to identify the cells expressing costimulatory molecules, without success.

Luteinizing hormone, PGF_2α _and TNF-α, affect progesterone secretion by cultured bovine mixed luteal parenchymal cells [15,34]. In addition, TNF-α has been shown to stimulate prostaglandin production in cultured bovine mixed luteal parenchymal cells [15,16] and also affects viability and gene expression in cultured bovine mixed luteal parenchymal cells [35,36]. However, LH, PGF_2α _and TNF-α, individually or in combination, were without effect on steady-state concentrations of CD80 and CD86 mRNA in cultured bovine mixed luteal parenchymal cells. Though initially interpreted as a negative result, these results may provide a subtle clue to the identity of cells expressing costimulatory molecules in the CL. Given the lack of effect of these known modulators of luteal parenchymal cell function on steady-state concentrations of costimulatory molecule mRNA in mixed luteal parenchymal cell cultures, one possible interpretation of these results is that the luteal parenchymal cells do not express costimulatory molecules. However, it is equally plausible that luteal parenchymal cells express costimulatory molecules, but that expression is simply not regulated by LH, PGF_2α _or TNF-α. Future studies are needed to conclusively determine whether bovine luteal parenchymal cells express costimulatory molecules.

A costimulatory signal is necessary for antigen presenting cells to induce activation and proliferation of T cells [30-32]. To determine whether functional costimulatory molecules are expressed on luteal parenchymal cells, T lymphocyte proliferation assays were performed in which freshly dissociated mixed luteal parenchymal cells from midcycle CL were placed in co-culture with T lymphocytes, as previously described [21,22]. In addition, PBMCs served as a control antigen presenting cell population in these studies, since monocytes/macrophages and B cells that express costimulatory molecules are present in PBMCs. Studies using antigen presenting cell lines have demonstrated that antibodies that bind to costimulatory molecules can interfere with costimulation and inhibit MHC-dependent T lymphocyte proliferation stimulated by antigen presenting cells *in vitro *[46-48]. In the present study, anti-bovine CD80 and anti-bovine CD86 mAbs inhibited mixed luteal parenchymal cell-stimulated T lymphocyte proliferation, and both antibodies used in combination exerted an inhibitory effect that appeared to be additive. Using a B cell lymphoma line Chen and coworkers [49] observed a similar effect, as was the case in the present study when PBMCs were used as antigen presenting cells. Therefore it can be concluded that the costimulatory molecules expressed by cells of the bovine corpus luteum are functional.

Cells within the bovine CL express both classes of MHC molecules. The presence of class I and class II MHC molecules on the surface of a cell allows the cell to interact with CD8+ and CD4+ T lymphocytes, respectively. In the cow, expression of class I MHC molecules does not vary with stage of the estrous cycle. Cells positive for class II MHC molecules are nearly undetectable in early CL, but expression increases by midcycle [20], indicating induction of class II expression that may coincide with the acquisition of luteolytic capacity. Further, numbers of cells positive for class II MHC molecules, and degree of class II expression by positive cells, both increase near the time of luteal regression, but are lower in pregnant compared to non-pregnant animals [20]. This pattern of expression suggests the involvement of MHC molecules in a mechanism that facilitates luteal regression. In this context, it is enigmatic that expression of costimulatory molecules is greatest during midcycle and declines near the time of luteal regression. It is possible that steady-state concentrations of mRNA for CD80 and CD86 are greatest during midcycle, but that protein concentrations are elevated toward the end of the estrous cycle. From the present studies it is not possible to draw conclusion about temporal expression of the cell surface proteins, but expression of functional costimulatory molecules on parenchymal cells isolated from midcycle CL has been conclusively demonstrated. It can therefore be inferred from the data from this and other studies that parenchymal cells from fully functional, mid-luteal phase bovine CL posses the capacity to stimulate T lymphocyte activation [21,22], due to expression of both class II MHC molecules [20] and expression of costimulatory molecules (present study). If MHC-mediated activation of T lymphocytes plays a role in luteal regression, it seems confounding that mixed luteal parenchymal cells from midcycle CL are able to stimulate proliferation of T lymphocytes. However, in a previous study we demonstrated that progesterone, which is present in very high concentrations in midcycle luteal tissue, inhibits luteal parenchymal cell-stimulated T lymphocyte proliferation [22]. The ability of cells within the CL to stimulate the activation of T lymphocytes may thus be attenuated by mediators present within the luteal microenvironment, modulating the activity of the lymphocytes in a manner dependent on the stage of the estrous cycle.

## Conclusion

This study demonstrates the presence of mRNA encoding the molecules CD80 and CD86 in bovine luteal tissue, as well as the presence of functional costimulatory molecules on luteal parenchymal cells. Costimulatory molecule mRNA is highest during midcycle, at a time when the CL is fully functional and progesterone production is maximal. Low concentrations of CD80 mRNA were found in luteal endothelial cell cultures, indicating that luteal endothelial cells may be a source of CD80 mRNA in the bovine CL, but contaminating macrophages cannot be ruled out as a source of CD80 mRNA in these cultures. Given the pattern of expression of costimulatory molecules in the CL, it seems unlikely that these molecules are involved in the process of luteal regression. However, the absence of costimulatory molecules on luteal endothelial cells expressing class II MHC antigens could provide a mechanism for inducing anergy in infiltrating T cells, thereby maintaining tolerance to the CL. Further studies are needed to examine the outcome of interactions between the various types of luteal parenchymal cells (steroidogenic, endothelial) and T lymphocytes, to determine how these interactions modulate T cell function.

## Authors' contributions

With the exception of the luteal endothelial cell isolation, culture, and RNA extraction, MJC collected all samples, conducted all experimental procedures described, and analyzed the data presented in the paper. Purification and culture of endothelial cells, and extraction of endothelial cell RNA were performed in the lab of JSD, who also contributed to preparation of the manuscript. JLP contributed to preparation of the manuscript and served as the PI on these experiments. Experiments were conceived of and designed by MJC and JLP.
